# Effects of Traditional Chinese Exercise Yijinjing on Disability and Muscle Strength Among Patients With Chronic Low Back Pain: Protocol for a Randomized Controlled Trial

**DOI:** 10.2196/67557

**Published:** 2025-05-07

**Authors:** Lei Guo, Cheng Wang, Xue Bai, Yukui Tian, Mengni Shi, Min Fang, Jing Xian Li, Qingguang Zhu, Junchang Liu

**Affiliations:** 1 College of Traditional Chinese Medicine Xinjiang Medical University Urumqi China; 2 Yueyang Hospital of Integrated Traditional Chinese Medicine and Western Medicine Shanghai University of Traditional Chinese Medicine Shanghai China; 3 School of Human Kinetics Faculty of Health Sciences University of Ottawa Ottawa, ON Canada

**Keywords:** chronic low back pain, traditional Chinese exercise, Yijinjing, muscle strength, randomized controlled trial

## Abstract

**Background:**

Chronic low back pain (CLBP) is a common public health problem. Progressive loss of muscle strength leads to long-term chronic pain and disability. Yijinjing exercises, an ancient therapy dating back thousands of years, are widely used in China to treat low back pain. However, little is known about its benefits and scientific evidence for back extensor strength. This trial aimed to assess the efficacy of Yijinjing on disability and dorsal extensor strength in patients with CLBP.

**Objective:**

We present a randomized controlled study to evaluate the efficacy of the traditional Chinese exercise Yijinjing on disability and back extensor strength in patients with CLBP.

**Methods:**

This is a 2-arm, parallel-design, assessor-blinded, and analyst-blinded randomized controlled trial. The 106 participants with CLBP who were recruited will first receive basic traditional Chinese manual therapy to help relieve their physical discomfort. Second, they will be randomly divided into a Yijinjing group (n=53) and a control group with functional exercises (n=53) at a ratio of 1:1. The interventions for both groups will be carried out twice a week for 4 weeks. Patients in both groups will be followed up at 1 and 3 months after the intervention. The primary outcome is disability (measured by the Oswestry Disability Index). The secondary outcomes included pain intensity (assessed by the Numerical Rating Scale), data from isokinetic dynamometry, flexibility (assessed by the fingertip-to-floor test), mood (evaluated by the Pain Catastrophizing Scale and Fear Avoidance Beliefs Questionnaire), and quality of life (measured by the EQ-5D-5L). All adverse effects will be assessed using the Treatment Emerging Symptoms Scale, and data will be analyzed using an intention-to-treat analysis.

**Results:**

The trial was funded in December 2023. The Institutional Ethics Committee of Yueyang Hospital of Integrative Medicine, Shanghai University of Traditional Chinese Medicine, approved this study. The first patient was enrolled in February 2024, and as of August 2024, a total of 106 participants have been recruited. Data analysis has not yet begun and is expected to be published in January 2025. The protocol has been registered with the Chinese Clinical Trial Registry (ChiCTR2400081105).

**Conclusions:**

If this trial proves effective, it will guide the setup of a randomized controlled trial to demonstrate whether traditional Chinese exercise Yijinjing improves disability in patients with CLBP and is more effective than usual stretching exercises.

**Trial Registration:**

Chinese Clinical Trial Registry ChiCTR2400081105; https://www.chictr.org.cn/showprojEN.html?proj=214425

**International Registered Report Identifier (IRRID):**

DERR1-10.2196/67557

## Introduction

Chronic low back pain (CLBP, Multimedia Appendix 1) is the second most common reason for medical consultations worldwide. It leads to disability and loss of muscle strength and affects quality of life and work [1,2]. This results in a high health care burden and indirect social costs [3]. Although most patients with CLBP recover quickly with a variety of medications, including nonsteroidal anti-inflammatory drugs, muscle relaxants, and opioids, 20%-30% still fail to improve function sufficiently, endure diminished lower back muscle strength, and even experience pain recurrence or worsening [4,5]. Therefore, active research into complementary therapies for CLBP to improve function, increase muscle strength, and reduce recurrence is important.

In China, many people with CLBP voluntarily seek traditional Chinese manual therapy (TCMT). It provides short-term pain relief. However, the recurrence rate is high. This is likely because TCMT is a passive therapy with limited effects on muscle strength recovery [6]. As popular sports become more influential in society, increasing attention is being given to the impact of physical activity on mental health [7,8]. Physical activity plays an important role in CLBP [9-11]. Nonpharmacological treatments recommended for CLBP include exercise therapy, yoga, and back school [5]. Among these treatments, yoga is widely recommended for patients with CLBP, but there are many types [12,13]. It is too difficult to choose for patients with CLBP. In China, many patients with CLBP voluntarily seek Yijinjing, which has a long history as an ancient traditional Chinese exercise therapy that originated in 221 BC. It combines positive thinking with deep contemplation, fluid movement, deep breathing, and relaxation techniques to promote the circulation of vital energy (or Qi) throughout the body [14]. It is considered a multifaceted intervention that includes physical, mental, social, emotional, spiritual, and cognitive-behavioral aspects. Our previous studies demonstrated that Yijinjing can significantly reduce the disability and pain associated with osteoarthritis and increase the effects on mood and muscle strength [15,16]. However, its potential benefits for CLBP remain less well studied [17]. Therefore, if proven beneficial for CLBP, Yijinjing could provide another exercise therapy option for the large number of Chinese residents enduring CLBP.

This RCT compared Yijinjing with self-stretching exercises (SSE), such as yoga. We hypothesized that patients randomized to Yijinjing would have greater short- and long-term improvements in functional disability, back muscle strength, and other outcomes associated with CLBP compared to those randomized to stretching training. We will explore the correlation between these outcomes.

## Methods

### Study Design

**Figure 1:**
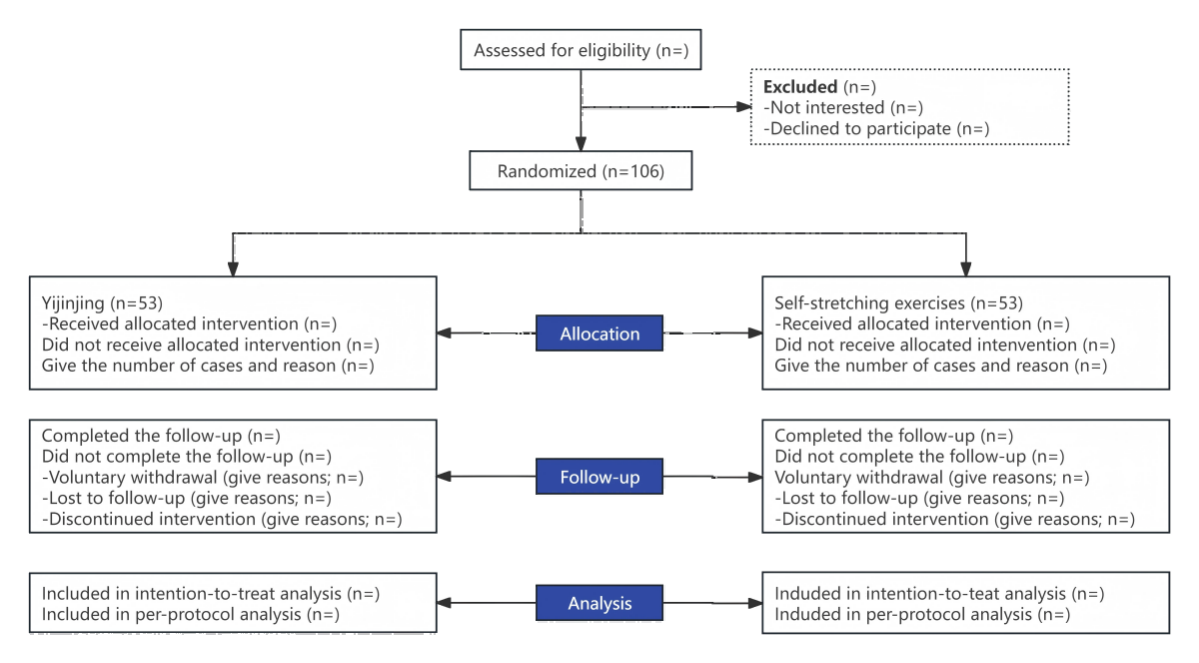
Flowchart of the study procedure.

This study is a single-center, assessor-blinded and analyst-blinded prospective randomized controlled trial with 2 parallel arms. This study was conducted in Shanghai, China, at Yueyang Hospital of Integrated Traditional Chinese and Western Medicine affiliated with Shanghai University of Traditional Chinese Medicine. All eligible participants will be recruited. First, they receive basic TCMT to help relieve their physical discomfort. Second, they will be randomly assigned to the Yijinjing and SSE groups at a 1:1 ratio after receiving TCMT. This trial protocol was approved by the Ethics Committee of Yueyang Hospital of Integrated Traditional Chinese and Western Medicine affiliated with the Shanghai University of Traditional Chinese Medicine (project number 2023-203). It is registered with the Chinese Clinical Trial Registry (ChiCTR2400081105). All patients signed a written informed consent form. The intervention will be administered twice per week for 1 month. The outcome assignment and statistical analyses will be completed blindly. The trial flowchart and research design are shown in Figure 1. The protocol for this study will adhere to rigorous standards as outlined by the Declaration of Helsinki, the CONSORT (Consolidated Standards of Reporting Trials) [18], and the SPIRIT (Standard Protocol Items: Recommendations for Interventional Trials) [19] guidelines. This commitment ensures the ethical and transparent conduct of our trial.

### Outcome

This study will evaluate the Oswestry Disability Index (ODI), Numerical Rating Scale (NRS), Pain Catastrophizing Scale (PCS), Fear Avoidance Beliefs Questionnaire (FABQ), EQ-5D-5L, the Flexibility Test, and the muscle strength test in the fourth week of the experiment. Furthermore, the results of the ODI, NRS, PCS, FABQ, and EQ-5D-5L will be followed up at the 8th and 16th weeks. The schedule of evaluations is outlined in Table 1 (Multimedia Appendix 2).

**Table 1:** Schedule of enrollment, intervention, and outcome measures.

Enrollment	Screening and allocation	Treatment period		Follow-up period	
	Week 0	Week 1-3	Week 4	Week 8	Week 16
Inclusion or exclusion criteria	✓	—^a^	—	—	—
Informed consent form	✓	—	—	—	—
Medical history	✓	—	—	—	—
Physical examination	✓	—	—	—	—
Eligibility for study	✓	—	—	—	—
Baseline characteristics	✓	—	—	—	—
C-spine x-ray	✓	—	—	—	—
Allocation	✓	—	—	—	—
Intervention	—	—	—	—	—
Yijinjing^b^	—	—	—	—	—
SSE^b,c^	—	—	—	—	—
Outcomes	—	—	—	—	—
ODI^d^	✓	—	✓	✓	✓
NRS^e^	✓	—	✓	✓	✓
PCS^f^	✓	—	✓	✓	✓
FABQ^g^	✓	—	✓	✓	✓
EQ-5D-5L	✓	—	✓	✓	✓
Flexibility test	✓	—	✓	—	—
Muscle strength test	✓	—	✓	—	—

^a^Not applicable.

^b^Two times a week from weeks 1-4.

^c^SSE: self-stretching exercises.

^d^ODI: Oswestry Disability Index.

^e^NRS: Numeric Rating Scale.

^f^PCS: Pain Catastrophizing Scale.

^g^FABQ: Fear Avoidance Beliefs Questionnaire.

### Participant Recruitment

Recruitment advertisements for this trial will be posted in the outpatient clinics of Yueyang Hospital and the Traditional Chinese Manipulative Therapy Department. Additionally, we will advertise through WeChat, which is the most widely used social media platform in China, to ensure easy access to interested participants. Potential participants will contact this study’s team by phone or WeChat. One of the researchers will verbally explain this study’s protocol and eligibility criteria to the potential participants and assess their eligibility over the phone after they provide verbal consent. Eligible potential participants will receive participant instructions and informed consent via WeChat or email, which will allow them at least 24 hours to review the documents. If the potential participant still expressed interest in participating and met the eligibility requirements, then they were invited to attend a baseline meeting. At baseline, one of our researchers will review this study’s protocol, reconfirm participant eligibility, and obtain written informed consent. Additionally, a baseline assessment will be conducted during the meeting, which will include all primary and secondary outcomes and demographic information. Thereafter, patients will be randomized.

### Sample Size Calculation

The calculations are based on hypothetical changes in the ODI at week 6. We use the results of a randomized controlled trial to formulate the intended effect. We hypothesize a 3.57-point improvement on the ODI scale in the Yijinjing group compared to the control group [20]. The sample size for each group provided 90% power, and these differences were detected using PASS software (version 15.0.5; NCSS, LLC) at a 2-sided significance level of .05. Considering an expected dropout rate of 20%, a total of 106 patients are required for this study.

### Eligibility Criteria

#### Inclusion Criteria

The inclusion criteria are (1) the duration of CLBP is at least 3 months [21], and CLBP can be accompanied by numbness and weakness in the lower limbs; (2) computed tomography or magnetic resonance imaging showing lumbar disc herniation; (3) age between 20 and 60 years, regardless of gender; (4) NRS≥3 points and persistent low back pain (LBP) for more than 12 weeks; (5) those who have not received any other treatment regimen regarding lumbar disc herniation within the last 1 month; (6) no long-term exercise training in the last 3 months, including swimming, jogging, and yoga; and (7) voluntarily participate in this trial and sign an informed consent form.

#### Exclusion Criteria

The exclusion criteria are (1) combined with other major lumbar diseases, such as tumors and tuberculosis; (2) history of spinal surgery within 1 year; (3) those who are scheduled to undergo lumbar surgery in the next 3 months or who have plans to go out for a long period; (4) patients with severe mental system disorders (eg, schizophrenia, bipolar disorder, and major depression); and (5) pregnant or lactating women.

#### Randomization

This trial used computer-generated block randomization with a fixed block size of 6 to ensure balanced group allocation. The intervention is unified after the block is full. Randomization sequences were securely stored in sequentially numbered, opaque, sealed envelopes to maintain allocation concealment. These envelopes were managed by an independent staff member not involved in trial procedures or participant assessments. Envelopes were opened sequentially after participant enrollment was formally confirmed, strictly following the order of study identification numbers assigned at recruitment. Each enrolled participant received a unique study identifier linked to their randomization group, ensuring traceability while preserving blinding integrity throughout this trial.

#### Blinding

Participants and therapists will not be blinded due to differences in treatment methods. Assessors, data managers, and analysts will not be aware of the group assignments in the outcome evaluation process and data analysis to reduce the risk of bias.

#### Interventions

In Chinese hospitals, manipulative therapy interventions are the first choice for patients with CLBP; therefore, manipulative therapy was chosen as the basic treatment protocol before randomization. It is administered by licensed doctors who have a minimum of 5 years of clinical experience in treating LBP. They have extensive experience in practicing manipulative therapy. In a previous study [22-24], we provided a detailed description of the manipulative therapy technique applied to CLBP. A similar technique will be used in this trial. A complete manipulative therapy intervention will have a duration of 15 minutes. Specifically, kneading and rolling techniques will be used to relax the muscles in the patient’s lower back. Palm root pressure was applied to the lower back muscles of patients with LBP, and pressure was applied along the erector spinae muscle from top to bottom. The pressure was applied for 10 minutes, 80-100 times per minute.

Once all patients were enrolled, baseline data were collected, and baseline information such as disease and age was recorded on the envelope to eliminate baseline imbalance and other factors in the randomization of blocks. After the manipulative therapy, all the patients will be randomly divided into 2 groups. Participants will receive 2 treatments every week for a total of 4 weeks. The teacher must pass clinical tests to ensure consistency in every treatment for all patients. At the same time, the participants may continue to take their usual pain relief medication, such as nonsteroidal anti-inflammatory drugs. Changes in dosage should be recorded on the case report form (CRF).

#### Yijinjing Group

**Figure 2:**
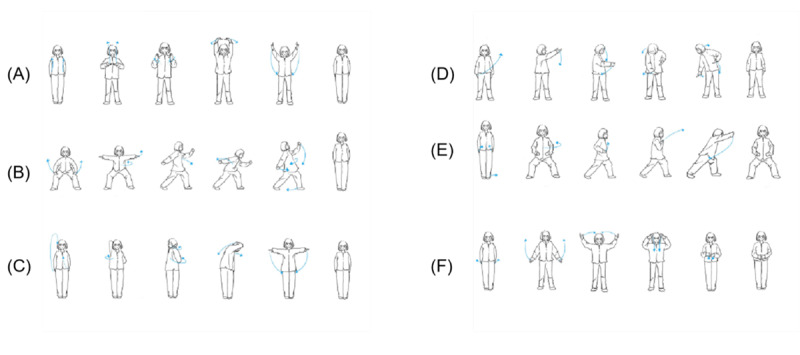
All movements of the 5-step program.

The participants in the Yijinjing exercise group will receive 4 weeks of Yijinjing exercise intervention. The course includes the instruction of a 5-step program, involving details such as the breathing rate, meditation method, extension rotation angle, and how to use your back muscles. Each session consisted of a warm-up for 5 minutes, 20 minutes of Yijinjing exercises, and 5 minutes of calming activities. The specific action characteristics are shown in Figure 2 and Table 2 (Multimedia Appendix 3) [25]. During the first week, the instructor guided the participants throughout the entire process, introducing the details and confusion to ensure that the correct exercise routine was followed. The teacher will provide ongoing support and supervision by videoconferencing. Participants were asked to record the time spent practicing in a punch card booklet, which was to be shown to the tutor. At the end of this trial, the participants were asked to return the booklet to the tutor so that the number of exercises could be counted.

**Table 2:** Details of the operation of the Yijinjing exercise. Specific information can be viewed on the Web [25].

Sequence of actions	Action details
Step 1: Resting the palm over the head	Take a step to the left. Raise your hands before your chest with palms up and fingertips facing. Rotate your wrists and raise your palms above your head. Slightly bend your elbow and look upward toward the dorsum of your palm. Lift your heels and stand on your toes. Hold for 5 s. Then, make fists, rotate your wrists, and slowly lower your fists to waist level. Place your whole feet on the ground. Repeat the entire procedure 3 times.
Step 2: Dragging the tail of a 9-cow ox backward	Begin by stepping sideways to the left with your left foot, while internally rotating both feet and slightly bending your knees and hips into a squatting position. Clench your fists and gradually lift them toward your chest. Extend your arms outward until they form a straight line on each side of your body. Rotate your torso to the left while lowering into a lunge position with your left leg bent and your right leg straight. Externally rotate your left forearm and flex your elbow to create a semicircular shape in front of your chest. Simultaneously, internally rotate your right forearm and extend it behind you. Keep your right elbow naturally extended and slightly backward at approximately 30°. Lastly, relax and repeat the sequence on the right side. This exercise should be performed symmetrically on both sides.
Step 3: Nine demons drawing their swords	Step to the left while crossing your hands over your chest and lifting them upward. Separate your hands above your head. With your left hand placed on your neck and your right hand on your back, tilt your head to the left at a 45° angle and twist your waist. Exhale while tightening your hands. Maintain this position for 5 s. Next, lower your hands to both sides of your body and relax as you exhale. Repeat the exercise on the right side.
Step 4: The green dragon probing the claws	Step your left foot sideways to the left while ensuring your feet are shoulder-width apart. Raise both palms upward to waist level while keeping your gaze forward. Extend your left palm diagonally forward and upward and reach above your head while simultaneously rotating your body to the right. Keep your right palm positioned by your waist on the right side to maintain stability. Internally rotate your left arm while turning your palm downward, and bend your torso from the right side while leaning toward the outer edge of your right foot. As you rotate your body to the left, trace a semicircle with your arm in front of your body. Inhale deeply as you gradually straighten your torso while lifting your hands from the right side and retracting them toward your body.
Step 5: A hungry tiger pouncing on its food	Start by positioning your feet shoulder-width apart. Rotate both feet to the left while turning your body in the same direction. Keep your palms facing upward as you lift both hands together until they reach chest level. Rotate your hands outward and lean your body forward while extending your hands forward with force. Arch your back forcefully and bend the front leg while straightening the back leg. Relax your arms, then turn your hands back into fists and slowly bring them down to the sides of your waist. Repeat this movement.
Step 6: Finishing moves	Open your feet as wide as your shoulders. Inhale and open your hands and raise them slowly above your head. Flip your palms. Exhale and press your hands downward and stop at your abdomen. Drop your hands down. Return to the initial preparation position.

#### About SSE

**Figure 3:**
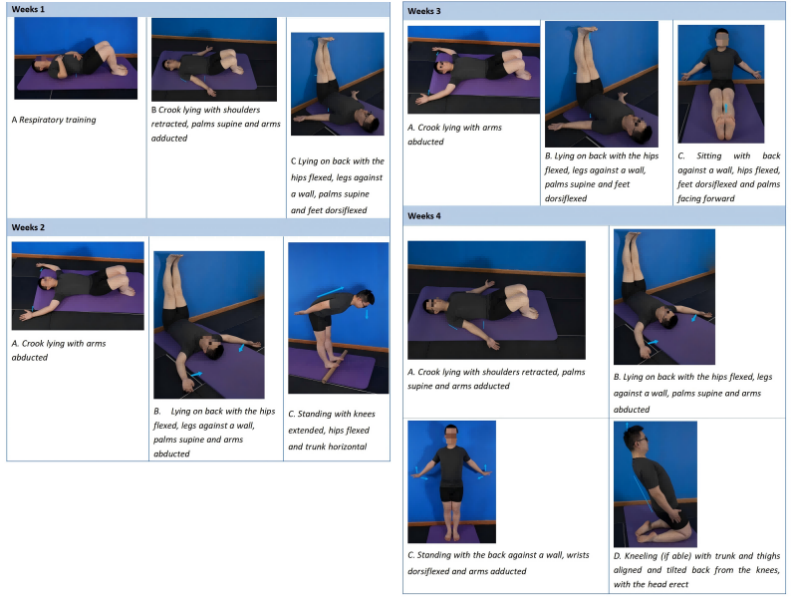
SSE protocol. SSE: self-stretching exercises.

Through graphic media, videos, and personal dialogue, participants received detailed explanations of biopsychosocial knowledge related to LBP. The educational content will aim to better understand pain, promote healthy work and lifestyle habits, and encourage moderate physical activity. These educational initiatives will continue throughout the intervention. This study will reference the previously validated SSE program used in published research for its effectiveness in treating a specific population with CLBP [26]. The therapist provided verbal cues and instructions for the stretching postures. Participants were instructed to engage in a warm-up for 5 minutes before the session, 20 minutes of SSE exercises, and a cool-down for 5 minutes afterward. Throughout the sustained stretching phase, participants were encouraged to advance each posture at their own pace. The primary goal was to achieve full back extension and maximum ankle dorsiflexion in each posture. Some postures targeted maximal back muscle abduction, while others focused on back muscle adduction. Participants were also advised to reduce the arching of their lower back (maintaining contact with the surface). Proper breathing techniques were emphasized during all postures, with participants instructed to inhale deeply through the nose, expanding the lower ribcage during inhalation. Exhalation involved lowering the chest and allowing the abdomen to expand. The specific action characteristics are shown in Figure 3 (Multimedia Appendix 4).

### Primary Outcome

The ODI is a disability questionnaire specific to the disease that consists of 10 items (scoring between 0 and 100) [27]. It is among the most frequently used in populations with LBP and is a fundamental measure of outcomes suggested for assessing disability related to pain in this group of patients. The validity, reliability, and responsiveness of the ODI have been demonstrated for individuals with LBP [28].

### Secondary Outcome

#### About NRS

The outcome was pain intensity, which was measured on an 11-point NRS (a score of 0 indicates no pain; 10 indicates the worst pain imaginable) 4, 8, and 16 weeks after the intervention [29,30].

#### Measures of Muscle Strength

**Figure 4:**
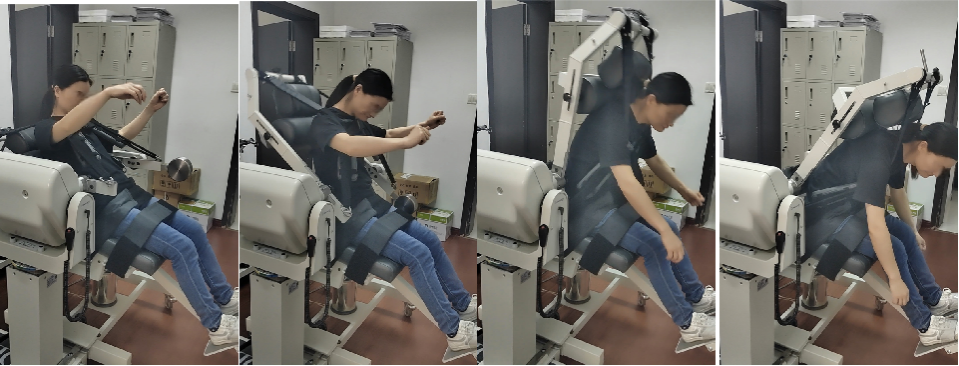
Biodex isokinetic muscle strength schematic diagram of the test method.

The maximal voluntary muscle strength of the extensor and flexor muscles will be measured by isokinetic dynamometry at angular velocities of 90 and 120 deg/s (Biodex System 4 Pro, Biodex Medical System) at baseline and during week 4. Subjects were advised to wear comfortable, loose-fitting clothing on the day of testing. Both the thighs and the backs of the subjects were fixed to the testing chair using straps. The axis of the dynamometer was located on the anterior superior iliac spine of the pelvis of the patient. During the formal test, each patient will be required to fold their arms across their chest and be given verbal encouragement in an attempt to achieve a maximum level of effort. Angular velocities of 90 and 120 deg/s will be applied to 2 sets of 5 maximal waist extensions and flexions with a rest interval of 30 seconds between sets [31]. The Biodex isokinetic muscle strength test method is shown in Figure 4.

#### About PCS

The PCS will be used for the assessment of catastrophizing thinking associated with chronic pain. It comprises 13 self-report items, and individual perception determines the total score, which ranges from 0 (indicating no catastrophizing) to 52 (indicating extremely severe catastrophizing). The PCS has demonstrated significant clinical acceptance, good construct validity, excellent internal consistency, and test-retest reliability [32,33].

#### About FABQ

The FABQ consists of 16 items, with items 1-5 measuring how fear avoidance beliefs about physical activity affect LBP and items 6-16 measuring how fear avoidance beliefs about work affect LBP, with each item rated on a scale of 0-6 (from “strongly disagree” to “strongly agree”) [34]. The FABQ has demonstrated robust psychometric properties, and its cross-cultural validity has been confirmed [35].

#### About EQ-5D-5L

The 5-level EQ-5D-5L Questionnaire will be used to evaluate health-related quality of life [36]. It encompasses 5 dimensions: mobility, self-care, usual activities, pain or discomfort, and anxiety or depression. Each dimension consists of 5 levels, which correspond to different points. Additionally, the EQ-5D-5L includes a visual analogue scale ranging from 0 (representing the worst imaginable health) to 100 (representing the best imaginable health) [37].

#### Flexibility

The fingertip-to-floor test is a global stretching assessment that was described and validated by Perret et al [38]. The participant stood barefoot on a 20 cm-high platform with their feet together, and the torso was bent forward as much as possible while the knees, arms, and fingers were extended for the test. The vertical distance between the tip of the middle finger and the platform should be measured with a flexible measuring tape, and the measurement is expressed in centimeters. A test was considered positive when the tip of the middle finger did not reach the platform and negative when it passed through the platform. This test has good psychometric properties [39]. The minimum clinically important difference has not been established, but the minimum detectable change for this test was 4.5 cm.

#### Dosage of Pain-Relieving Medication

The participants may continue to take their usual pain relief medication, such as nonsteroidal anti-inflammatory drugs. Changes in dosage should be recorded on the CRF. Dose diaries are notebooks in which participants record their daily dose of pain medication as well as the duration of the dose. The researcher does not recommend any changes in the medical regimen.

### Data Collection and Monitoring

The data collectors, who are blinded to the subgroups and not involved in other aspects of this study, will accurately record all the data collected into the CRF. Upon completion of this trial, the CRF will be handed over to 2 professional data workers who are also blinded to allocation and who are not involved in other parts of this study. The 2 data workers independently extract the data from the CRF into Excel (Microsoft Corp) and verify the accuracy of the data. All electronic data will be stored on a password-protected server, while all paper data will be kept in a locked filing cabinet. Additionally, deidentified data will be stored separately from the files and filing cabinets containing participant details and trial identification numbers. An electronic data capture system will also be used throughout this study to record any traces of data entry or modification. The Department of Information Science and the Clinical Research Centre of Yueyang Hospital, Shanghai University of Traditional Chinese Medicine, will provide the electronic data capture platform and will oversee the entire process of study data collection and management. They will review this study’s data every 6 months to ensure the quality of this study and the reliability of the data.

### Statistical Analysis

In this study, the principle of intentionality analysis will be used to analyze the data. The safety analysis set will be used to analyze the safety of this study. A statistician who is unaware of the group assignments will use SPSS statistical software (version 26.0; IBM Corp) to analyze the data. All effects will be estimated using 95% CIs, and all the statistical tests will be 2-sided. A *P* value less than .05 indicated statistical significance.

Continuous variables are presented as the mean (SD) if they follow a normal distribution; otherwise, they are presented as medians and quartiles. Categorical variables are presented as percentages. Primary outcomes will be assessed using analysis of covariance adjusted for baseline. For other secondary outcomes, *t*-tests or Wilcoxon rank-sum tests will be used.

Repeated outcomes over time will be compared using linear mixed-effects models with the following fixed effects: (1) time points (categorical), (2) intervention group, (3) time-by-group interaction, and (4) baseline measurement as a covariate. Random intercepts for participant ID will be included to account for within-subject correlations. Additional adjustments will be made for prespecified covariates, including age, gender, and study site (if multicenter). Bonferroni correction was used for multiple comparisons across time points.

Missing data will be replaced according to the principle of carry-over of last observations. Missing values for dropout cases will be imputed using either the last observation carried forward method if the dropout rate is below 5% or the multiple imputation method for a dropout rate of 5% or higher. If the attrition rate exceeds 10%, we use per-protocol analysis to ensure the reliability of this study.

### Research Security and Quality Control

Quality control will be conducted during this trial under the management of the steering committee. Before researchers participate in this trial, professional trial methods and regular monitoring techniques should be used to ensure the consistency of the methods. If this study protocol is modified or corrected, then the steering committee and ethics committee should be informed. Quality control will be performed during this trial under the management of the steering committee. Researchers should be trained in specialized test methods and routine monitoring techniques before participating in this trial to ensure consistency of the approach. The Steering Committee and Ethics Committee were informed of any modifications or corrections to this study’s protocol. During this study, a booklet was given to each patient, and patients were asked to record the time and number of exercises per day.

The Ethics Committee of Yueyang Hospital of Integrative Medicine, Shanghai University of Traditional Chinese Medicine, will be responsible for the safety and quality control of this study. They may suggest changes in this study’s design to protect the participants, and the principal investigator will make the final decision. All the participants must sign the informed consent form before enrollment.

The following adverse events may occur in this trial. If participants report severe pain during treatment and follow-up and have a visual analog scale score greater than 8, they will be allowed to return to the hospital for another visit. This visit will be recorded in the CRF. If anything out of the expected range occurs, the therapist must report it to the investigator within 24 hours of the event, and the investigator will report it to the Safety and Treatment Monitoring Committee for recording.

### Ethical Considerations

This study has been approved by the Medical Ethics Committee of Yueyang Hospital of Integrated Traditional Chinese and Western Medicine affiliated with Shanghai University of Traditional Chinese Medicine (2023-203), and will be conducted per the guidelines and regulations approved by the participating institution. This trial is being conducted based on a protocol registered with the China National Clinical Trial Registry. Informed consent forms were obtained from all patients before enrollment in this study. Participation is voluntary, based on the patients’ wishes, and therefore, this study can be stopped at any time. All records containing names or other personal identifiers are stored separately from the research records identified by code numbers. Participants cannot be identified from any images in this paper or supplementary materials. The intervention method does not incur additional costs for participants; therefore, no compensation is required.

## Results

Our protocol has been successfully approved by the institutional review board of Yueyang Integrated Traditional Chinese and Western Medicine Hospital affiliated with Shanghai University of Traditional Chinese Medicine, obtaining ethical approval in December 2023, and registered with the Chinese Clinical Trial Registry (ChiCTR2400081105). As of August 2024, we have recruited 106 participants. Data analysis has not yet begun and is expected to be published in January 2025. At the time of submission, recruitment for this trial started. The first participant was included on February 20, 2024.

## Discussion

### Principal Findings

This study aims to provide critical evidence to support the therapeutic efficacy of Yijinjing intervention for CLBP. There is a lack of high-quality clinical trials on the effects of Yijinjing in CLBP, although we found some clinical evidence of this approach in patients, we hypothesize that a 12-week Yijinjing regimen will yield clinically meaningful improvements in functional disability, lumbar muscle strength, and pain intensity, with durable therapeutic effects persisting through a 3-month follow-up period. Crucially, concurrent improvements in emotional distress measures and lumbar flexibility would suggest that Yijinjing confers multidimensional benefits extending beyond biomechanical adaptations—a finding that could redefine its role as a holistic intervention addressing both somatic and psychosocial dimensions of CLBP. While existing literature substantiates the moderate efficacy of exercise therapies in CLBP management [40], Yijinjing’s distinctive focus on graded neuromuscular control and dynamic muscular equilibrium [41] differentiates it from conventional strength training paradigms, potentially offering unique mechanistic advantages.

This study’s strengths include multidimensional outcome assessment (functional, physiological, and psychological domains) and longitudinal follow-up. However, 3 limitations warrant acknowledgment: first, inherent challenges in participant blinding for behavioral interventions may introduce expectation bias in self-reported outcomes, though objective biomechanical measurements partially mitigate this concern. Second, the single-center recruitment strategy may constrain population generalizability. Third, while the pragmatic 30-minute intervention protocol enhances clinical translatability, it precludes exploration of dose-response relationships relative to extended regimens.

In conclusion, this trial will systematically evaluate Yijinjing’s efficacy and safety profile in CLBP management. Its findings may inform clinical guidelines for integrating traditional mind-body exercises into contemporary pain rehabilitation frameworks. Nevertheless, given the constrained intervention duration inherent to this design, subsequent investigations should prioritize extended treatment phases and prolonged follow-up windows to elucidate long-term outcomes and potential decay patterns of therapeutic benefits.

## Appendix

Multimedia Appendix 1 Abbreviations.

Multimedia Appendix 2 Schedule of enrollment, intervention, and outcome measures.

Multimedia Appendix 3 Details of the operation of Yijinjing exercise.

Multimedia Appendix 4 Self-stretching exercises (SSE) protocol.

## Abbreviations

CLBP: chronic low back pain
CONSORT: Consolidated Standards of Reporting Trials
CRF: case report form
FABQ: Fear Avoidance Beliefs Questionnaire
LBP: low back pain
NRS: Numerical Rating Scale
ODI: Oswestry Disability Index
PCS: Pain Catastrophizing Scale
SPIRIT: Standard Protocol Items: Recommendations for Interventional Trials
SSE: self-stretching exercise
TCMT: traditional Chinese manual therapy
