# Supplementary material for: Effects of Traditional Chinese Exercise Yijinjing on Disability and Muscle Strength Among Patients With Chronic Low Back Pain: Protocol for a Randomized Controlled Trial
Source: JMIR Res Protoc. 2025 May 7;14:e67557. doi: 10.2196/67557 (PMC12096028; doi:10.2196/67557)
Supplement: Multimedia Appendix 1 [file resprot_v14i1e67557_app1.docx]

| **Table S1.** Abbreviations. | |
| --- | --- |
| CLBP | Chronic low back pain |
| TJJ | YiJinJing |
| SSE | self-stretching exercises |
| Traditional Chinese Manual Therapy | TCMT |
| Oswestry Disability Index | ODI |
| numerical rating scale | NRS |
| Pain Catastrophising Scale | PCS |
| Fear Avoidance Beliefs Questionnaire | FABQ |
| The 5-Level EuroQoL-5 Dimensions Questionnaire | EQ-5D-5L |
| Case Report Form | CRF |
| electronic data capture | EDC |
| Traditional Chinese Medicine | TCM |
