# Supplementary material for: Effects of Traditional Chinese Exercise Yijinjing on Disability and Muscle Strength Among Patients With Chronic Low Back Pain: Protocol for a Randomized Controlled Trial
Source: JMIR Res Protoc. 2025 May 7;14:e67557. doi: 10.2196/67557 (PMC12096028; doi:10.2196/67557)
Supplement: Multimedia Appendix 2 [file resprot_v14i1e67557_app2.docx]

| **Table S1.** Schedule of enrollment, intervention, and outcome measures. | | | | | |
| --- | --- | --- | --- | --- | --- |
|  | **Enrolment and allocation** | | | | |
|  | **Screening/Allocation** | **Treatment period** | | **Follow-up period** | |
| **Time point** | **Week 0** | **Week 1-3** | **Week 4** | **Week 8** | **Week 16** |
| **Enrolment** |  |  |  |  |  |
| Inclusion/exclusion criteria | × |  |  |  |  |
| Informed consent form | × |  |  |  |  |
| Medical history | × |  |  |  |  |
| Physical examination | × |  |  |  |  |
| Eligibility for study | × |  |  |  |  |
| Baseline characteristics | × |  |  |  |  |
| C-spine X-ray | × |  |  |  |  |
| Allocation | × |  |  |  |  |
| Intervention |  |  |  |  |  |
| YJJ |  | Two times a week | |  |  |
| SSE |  | Two times a week | |  |  |
| Outcomes |  |  |  |  |  |
| ODI | × |  | × | × | × |
| NRS | × |  | × | × | × |
| PCS | × |  | × | × | × |
| FABQ | × |  | × | × | × |
| EQ-5D-5L | × |  | × | × | × |
| Flexibility Test | × |  | × |  |  |
| muscle strength test | × |  | × |  |  |
| ·Yijinjing (YJJ); Self-stretching Exercises (SSE); ODI (Oswestry Disability Index); NRS (Numeric Rating Scale); PCS (Pain Catastrophizing Scale); FABQ (Fear Avoidance Beliefs Questionnaire), EQ-5D-5L (5D-5L European Quality of Life Scale) | | | | | |
