# Supplementary material for: Effects of Traditional Chinese Exercise Yijinjing on Disability and Muscle Strength Among Patients With Chronic Low Back Pain: Protocol for a Randomized Controlled Trial
Source: JMIR Res Protoc. 2025 May 7;14:e67557. doi: 10.2196/67557 (PMC12096028; doi:10.2196/67557)
Supplement: Multimedia Appendix 3 [file resprot_v14i1e67557_app3.docx]

| **Table S1.** Details of the operation of Yijinjing exercise. | |
| --- | --- |
| Sequence of actions | Action details |
| Step 1:  Resting the palm over the head | Take a step to the left, raise your hands in front of your chest with palms up and fingertips facing. Rotate your wrists and raise your palms above your head. Slightly bend your elbow and look upwards towards the dorsum of your palm. Lift your heels and stand on your toes. Hold for 5 s. Then, make fists, rotate your wrists and slowly lower your fists to waist level. Place your whole feet on the ground. Repeat the entire procedure three times. |
| Step 2:  Dragging the tail of a nine-cow ox backwards | Begin by stepping sideways to the left with your left foot, while internally rotating both feet and slightly bending your knees and hips into a squat position. Clench your fists and gradually lift them towards your chest. Extend your arms outwards until they form a straight line on each side of your body. Rotate your torso to the left while lowering into a lunge position with your left leg bent and your right leg straight. Externally rotate your left forearm and flex your elbow to create a semi-circular shape in front of your chest. Simultaneously internally rotate your right forearm and extend it behind you. Keep your right elbow naturally extended and slightly backwards at approximately 30°. Lastly, relax and repeat the sequence on the right side. This exercise should be performed symmetrically on both sides. |
| Step 3:  Nine demons drawing their swords | Step to the left while crossing your hands over your chest and lifting them upwards. Separate your hands above your head. With your left hand placed on your neck and your right hand on your back, tilt your head to the left at a 45° angle and twist your waist. Exhale while tightening your hands. Maintain this position for 5 s. Next, lower your hands to both sides of your body and relax as you exhale. Repeat the exercise on the right side. |
| Step 4:  The green dragon probing the claws | Step your left foot sideways to the left while ensuring your feet are shoulder-width apart. Raise both palms upwards to waist level while keeping your gaze forwards. Extend your left palm diagonally forwards and upwards and reach above your head while simultaneously rotating your body to the right. Keep your right palm positioned by your waist on the right side to maintain stability. Internally rotate your left arm while turning your palm downwards, and bend your torso from the right side while leaning towards the outer edge of your right foot. As you rotate your body to the left, trace a semi-circle with your arm in front of your body. Inhale deeply as you gradually straighten your torso while lifting your hands from the right side and retracting them towards your body. |
| Step 5:  A hungry tiger pouncing on its food | Start by positioning your feet shoulder-width apart. Rotate both feet to the left while turning your body in the same direction. Keep your palms facing upwards as you lift both hands together until they reach chest level. Rotate your hands outwardly and lean your body forwards while extending your hands forwards with force. Arch your back forcefully and bend the front leg while straightening the back leg. Relax your arms, then turn your hands back into fists and slowly bring them down to the sides of your waist. Repeat this movement. |
| Step 6:  Finishing moves | Open your feet as wide as your shoulders. Inhale and open your hands and raise them slowly above your head. Flip your palms. Exhale and press your hands downwards and stop at your abdomen. Drop your hands down. Return to initial preparation position. |
| Specific information can be viewed by clicking on the link：https://mp.weixin.qq.com/s/lEOGNLvUc9MLkNAvV9SyWw | |
