# Supplementary material for: Effects of Traditional Chinese Exercise Yijinjing on Disability and Muscle Strength Among Patients With Chronic Low Back Pain: Protocol for a Randomized Controlled Trial
Source: JMIR Res Protoc. 2025 May 7;14:e67557. doi: 10.2196/67557 (PMC12096028; doi:10.2196/67557)
Supplement: Multimedia Appendix 4 [file resprot_v14i1e67557_app4.docx]

| **Table S1.** Self-stretching exercises (SSE) protocol. | | | |
| --- | --- | --- | --- |
| **Weeks 1** | | | |
| *A. Respiratory training*  *B. Crook lying with shoulders retracted, palms supine and arms adducted*  *C. Lying on back with the hips flexed, legs against a wall, palms supine and feet dorsiflexed* | | | |
| 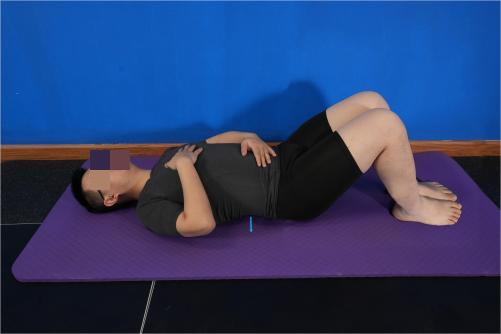A | | 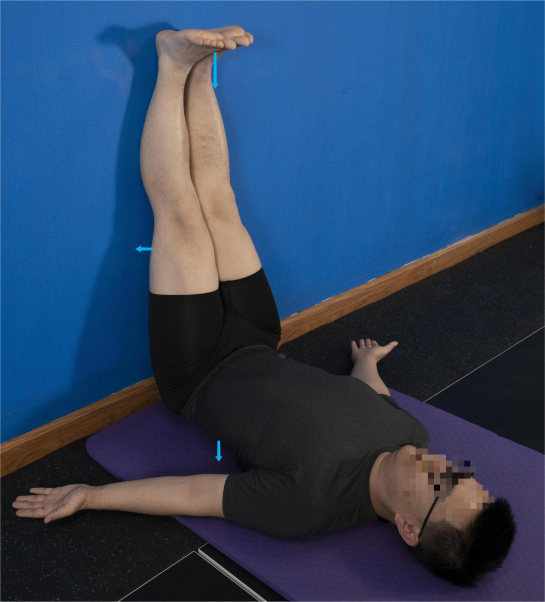  C | |
| 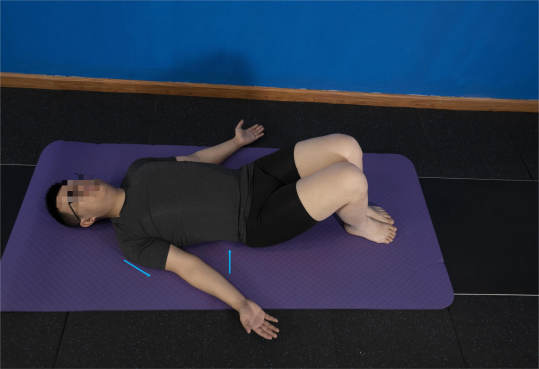B | |  |  |
| **Weeks 2** | | | |
| *A. Crook lying with arms abducted*  *B. Lying on back with the hips flexed, legs against a wall, palms supine and arms abducted*  *C. Standing with knees extended, hips flexed and trunk horizontal* | | | |
| 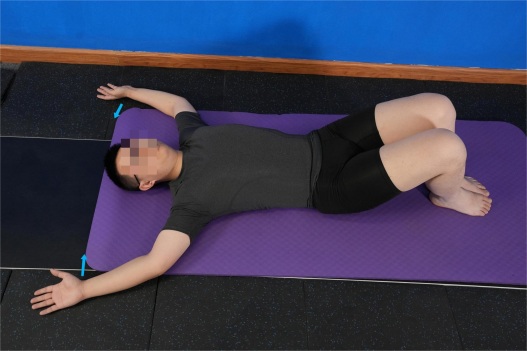  A | 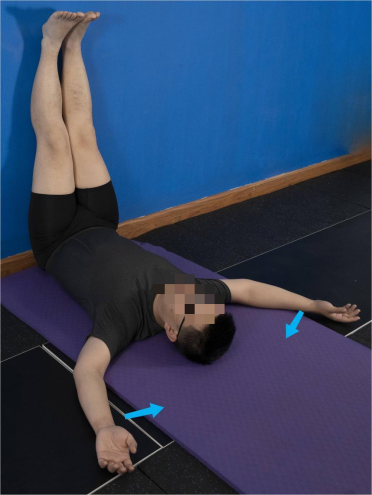B | | 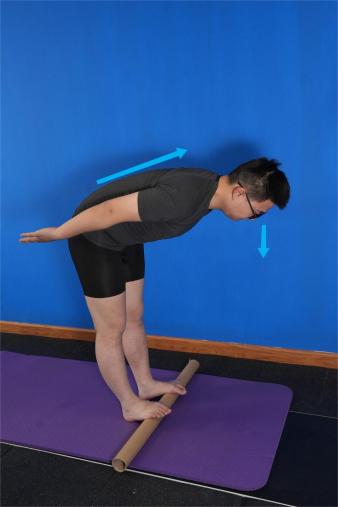C |

| **Weeks 3** | | | |
| --- | --- | --- | --- |
| *A. Crook lying with arms abducted*  *B. Lying on back with the hips flexed, legs against a wall, palms supine and feet dorsiflexed*  *C. Sitting with back against a wall, hips flexed, feet dorsiflexed and palms facing forward* | | | |
| 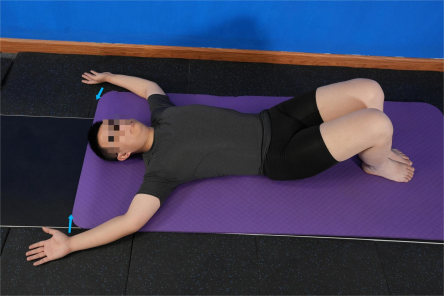  A | 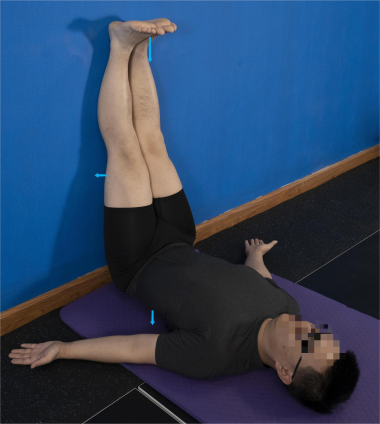B | | 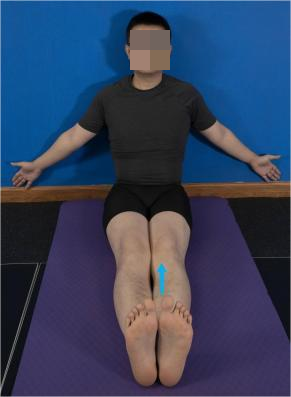C |
| **Weeks 4** | | | |
| *A. Crook lying with shoulders retracted, palms supine and arms adducted*  *B. Lying on back with the hips flexed, legs against a wall, palms supine and arms abducted*  *C. Standing with the back against a wall, wrists dorsiflexed and arms adducted*  *D. Kneeling (if able) with trunk and thighs aligned and tilted back from the knees, with the head erect* | | | |
| 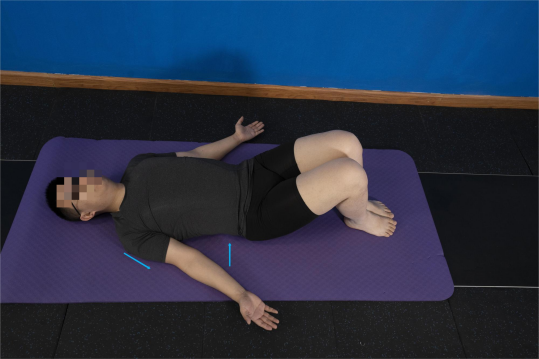A | | 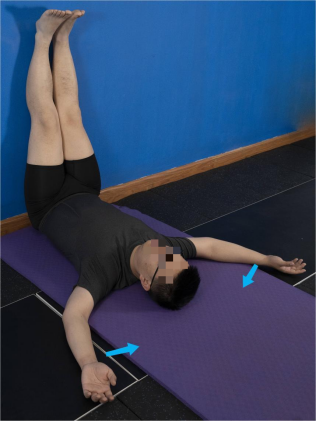B | |
| 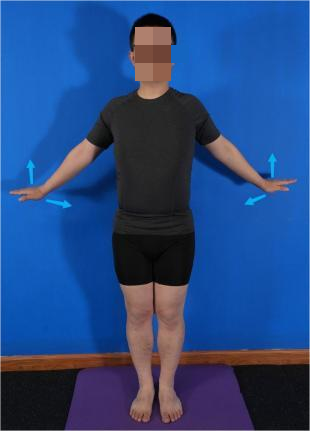C | | 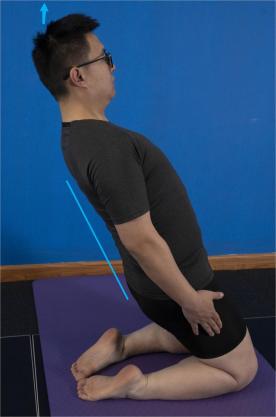D | |
| Specific information can be viewed by clicking on the link：https://mp.weixin.qq.com/s/qLK664MKNFv4mfQCRtA7Bg | | | |
